# Modeling the Maturation of Grip Selection Planning and Action Representation: Insights from Typical and Atypical Motor Development

**DOI:** 10.3389/fpsyg.2016.00108

**Published:** 2016-02-09

**Authors:** Ian Fuelscher, Jacqueline Williams, Kate Wilmut, Peter G. Enticott, Christian Hyde

**Affiliations:** ^1^Cognitive Neuroscience Unit, School of Psychology, Deakin UniversityMelbourne, VIC, Australia; ^2^College of Sport and Exercise Science, Institute of Sport Exercise and Active Living, Victoria UniversityMelbourne, VIC, Australia; ^3^Faculty of Health and Life Sciences, Oxford Brookes UniversityOxford, UK

**Keywords:** action planning, end state comfort, motor imagery, action representation, developmental coordination disorder

## Abstract

We investigated the purported association between developmental changes in grip selection planning and improvements in an individual’s capacity to represent action at an internal level [i.e., motor imagery (MI)]. Participants were groups of healthy children aged 6–7 years and 8–12 years respectively, while a group of adolescents (13–17 years) and adults (18–34 years) allowed for consideration of childhood development in the broader context of motor maturation. A group of children aged 8–12 years with probable DCD (pDCD) was included as a reference group for atypical motor development. Participants’ proficiency to generate and/or engage internal action representations was inferred from performance on the hand rotation task, a well-validated measure of MI. A grip selection task designed to elicit the end-state comfort (ESC) effect provided a window into the integrity of grip selection planning. Consistent with earlier accounts, the efficiency of grip selection planning followed a non-linear developmental progression in neurotypical individuals. As expected, analysis confirmed that these developmental improvements were predicted by an increased capacity to generate and/or engage internal action representations. The profile of this association remained stable throughout the (typical) developmental spectrum. These findings are consistent with computational accounts of action planning that argue that internal action representations are associated with the expression and development of grip selection planning across typical development. However, no such association was found for our sample of children with pDCD, suggesting that individuals with atypical motor skill may adopt an alternative, sub-optimal strategy to plan their grip selection compared to their same-age control peers.

## Introduction

In general terms, motor planning reflects the process of selecting a movement plan from an infinite number of solutions in order to achieve a desired end-state (Rosenbaum et al., 1992). Though often implicit, the nervous system must make this decision with consideration of the surrounding environment, involvement of external agents and the biomechanical constraints of relevant limbs. Indeed, neuro-computational modeling suggests that the intended movement, including its terminal location, may be implicitly simulated via internal (neural) representations of the forthcoming action prior to movement execution. In doing so, the most appropriate movement plan can be selected with consideration of a hierarchy of cost constraints to ensure that the goal of the forthcoming action is achieved in a comfortable and efficient manner (Rosenbaum et al., 1995, 2001, 2014; Flanagan and Wing, 1997; Johnson, 2000; Johnson et al., 2002; Glover, 2004; Johnson-Frey et al., 2004). Specifically, to simulate the intended action, the central nervous system is thought to use an efferent copy of the impending motor command (i.e., internal action representations) to predict the sensory consequences of an action given the current and the desired end-state of the limb (Wolpert and Kawato, 1998; Johnson, 2000; Wolpert and Ghahramani, 2000; Glover, 2004). At a neural level, this view is supported by recent imaging data which suggests that this process may call upon similar systems that are active during movement preparation, including parietal-cerebellar structures and premotor cortices (see Hétu et al., 2013 for a review).

Experimentally, this broad process can be observed using ‘grip selection’ tasks, which elicit the ‘end-state comfort’ (ESC) effect (Rosenbaum et al., 1990). These generally require an individual to grasp and manipulate an object such that an implicit decision must be made whether to sacrifice initial limb comfort in order to end movement in a comfortable position (i.e., ESC), or vice versa. Interestingly, healthy adults show an overwhelming tendency to opt for ESC during these tasks, an effect that has been reproduced across multiple paradigms (see Hermens et al., 2014). Furthermore, the ESC effect appears to strengthen as a product of typical development and is known to be reduced in a number of developmental disorders where motor impairment is a central feature (e.g., cerebral palsy; Mutsaarts et al., 2007; Crajé et al., 2010; Steenbergen et al., 2013). Accordingly, it is generally accepted that the strength of the ESC effect reflects the proficiency of grip selection planning, with stronger effects considered optimal (see Wunsch et al., 2013).

From a developmental perspective, the ESC effect appears to emerge in the pre-school years (i.e., 3–5 years of age), though the preference remains relatively weak and inconsistent when considered as a percentage of trials (see Rosenbaum et al., 2012 for a review). There is mounting evidence that the strength of the effect amplifies considerably during the primary school years. Specifically, around the age of 8, typically developing children show a substantial increase in the percentage of reach to grasp movements that terminate in ESC (Stöckel et al., 2012; Wilmut and Byrne, 2014a; Wunsch et al., 2014), with more subtle increases reported into later childhood and adolescence (Stöckel et al., 2012; Scharoun and Bryden, 2014; Wilmut and Byrne, 2014a). Since current computational modeling proposes that the type of grip-selection planning necessary for achieving ESC with consistency requires one to internally predict the sensory consequences of the end-state of the movement via internal action representations, a number of studies have suggested that the non-linear increases in the tendency to opt for ESC observed across typical development may be subserved, at least partly, by an improved ability to engage internal action representations (Toussaint et al., 2013; Noten et al., 2014; Wilmut and Byrne, 2014a).

### Developmental Changes in Planning for Grip Selection Coincide with Improved Action Representation in Typically Developing Children

Support for the view that developmental improvements in the proficiency of grip-selection planning during the primary school years (i.e., 6–12 years) and beyond may be associated with an improved capacity to represent action internally comes from motor imagery (MI) studies. These indicate that a child’s capacity to engage action representations follows a markedly similar developmental trajectory to that of grip selection planning. Briefly, MI refers to the imagination of a movement without any form of overt movement taking place (Jeannerod, 1994; Decety and Grèzes, 1999). Performance has been shown to conform to the same postural and biomechanical constraints as real action (see Munzert et al., 2009). Further, neuroimaging studies indicate that this relative functional equivalence is coupled with neurophysiological similarities, with actual and imagined movements activating similar neural pathways (Jeannerod, 2001; Munzert et al., 2009). Accordingly, it is argued that MI provides insight into the integrity of internal ‘neural’ action representations that precede and support overt movements, only brought to conscious awareness because the motor command has been inhibited (Jeannerod, 2001; de Lange et al., 2008; Munzert et al., 2009).

While a wide variety of MI measures are available, the hand rotation task has been one of the more favored measures for studies involving children (see Butson et al., 2014). Here, participants are required to make laterality judgments about single hands presented at varying angular rotations on a monitor. While children and adults commonly report imagining their own hand to make laterality decisions, the use of MI is inferred behaviourally by confirming that task performance conforms to the biomechanical and postural constraints of real action (see Fuelscher et al., 2015a,b). Similarly to the maturational properties of grip-selection planning, a number of studies using the hand rotation task suggest a critical developmental period for one’s ability to engage internal action representations during the primary/elementary school years (Caeyenberghs et al., 2009; Gabbard, 2009; Butson et al., 2014). This is then followed by a more incremental improvement into adolescence and adulthood (Conson et al., 2013; Fuelscher et al., 2015a). However, none of these developmental studies of MI also measured grip selection planning in their samples. Accordingly, it is difficult to determine the degree to which these improvements in MI (and hence the quality of internal action representations) may, or may not, be associated with the development of grip-selection planning.

### Are Developmental Improvements in Grip Selection Planning Associated with Internal Action Representations?

Given the theoretical association between the integrity of internal action representations and one’s capacity to plan their grip selection, it is perhaps surprising that only a single study of typical development has, to date, tested this association directly. Recently, Toussaint et al. (2013) explored motor planning in groups of healthy 6 and 8 year olds using a custom developed bar transport task designed to elicit the ESC effect. Correlations between the percentage of trials ending in ESC and performance on the hand rotation task indicated that, in each age group, more efficient hand rotation performance was associated with an increased propensity to plan for ESC on the bar grasping task; though the association fell just short of significance in the 8 year-old group. While these results provide preliminary evidence that the ability to engage internal action representations may be associated with the integrity of planning for grip selection in school-aged children, the limited age range does not allow one to infer developmental trends in either action representation or planning across the critical primary years (or beyond), nor how this proposed association evolves during late childhood and into adulthood.

The notion that successful grip selection planning may be dependent on one’s ability to engage internal action representations is further supported by evidence that children and adults with atypical motor skill (viz Developmental Coordination Disorder - DCD) show a reduced capacity for performing MI (Gabbard and Bobbio, 2011; Adams et al., 2014) and often demonstrate a decreased tendency to opt for ESC on grip selection tasks (van Swieten et al., 2010; Wilmut and Byrne, 2014b). In a recent study, Noten et al. (2014) were the first to investigate the extent to which motor planning in children with DCD was associated with action representation. Here, the authors compared performance of 7–12 years old children with and without DCD on a bar grasping task and on the hand rotation task. Consistent with earlier evidence on DCD, they found a reduced ability to engage in MI. However, no group differences in the tendency to adopt ESC were found. As noted by the authors, however, a ceiling effect was observed on the bar grasping task adopted, making it difficult to determine whether the non-significant difference reflected a genuine lack of difference in planning ability between DCD and control groups, or was a task-specific effect. Critically, contrary to earlier evidence reported in typically developing children, Noten et al. (2014) failed to detect significant correlations between the MI and end-state planning measures for either the DCD or control groups. This suggests no direct association between the quality of internal action representations and ones ability to plan for reach to grasp actions. However, if a true relationship did indeed exist, the previously noted ceiling effect on the bar grasping task would have decreased co-variability across the imagery and planning metrics, subsequently reducing the correlation co-efficient. In short, we argue that while the work of Noten et al. (2014) provides important preliminary insight into the association between the integrity of action representations and grip selection planning in DCD, the lack of task complexity on the bar grasping task and the metrics adopted to test the association limits the degree to which this association can be clearly elucidated in atypical, or typical, development.

### Summary

To summarize, there is a strong theoretical basis for the argument that the non-linear improvements in grip selection planning that characterize typical motor development may be associated with an increased capacity to represent action internally. However, few studies have directly tested this hypothesis, with those available either adopting a restricted developmental age-range or reporting ceiling effects that reduce the scope for probing this important relationship. Accordingly, it is difficult to verify the degree to which the previously established pattern of substantially improved grip selection planning that occurs during childhood (i.e., 6–12 years), and subsequent incremental improvements into adulthood are, or are not, associated with an individual’s capacity to generate and/or engage internal action representations. This knowledge, however, is critical to our understanding of the development of grip selection planning (and motor planning more generally), including the neurocognitive mechanisms that support it and its pathology.

Accordingly, the aim of this study was to test for a predictive association between developmental improvements in motor planning and the integrity of action representations. A group of children with probable DCD (pDCD) aged 8–12 years were included as a reference group for atypical motor development. The integrity of grip selection planning was measured using Wilmut and Byrne’s (2014a, 2014b) grip selection task, which includes movements of varying levels of complexity and has been shown to differentiate between children and adults with DCD and their peers without the presence of ceiling effects. The ability to generate internal action representations was measured using the well-validated hand rotation task. As per previous research, it was predicted that the development of grip selection planning would show a non-monotonic progression in healthy individuals, characterized by substantial increases in the tendency to plan for ESC from 6 to 12 years, with more incremental increases expected thereafter into adolescence and adulthood. Critically to the present paper, based on neuro-computational modeling indicating that internal action representations are associated with the expression and development of grip selection planning, regression analysis was expected to show that the predicted pattern of non-linear increases in the strength of the ESC effect through typical development was associated with increases in the efficiency of MI performance on the hand rotation task. A similar association was predicted in children with pDCD.

## Materials and Methods

### Participants

The sample consisted of 95 participants from which eight 6–7 year olds, two 8–12 year olds (one typically developing and one child with pDCD) and one 13–17 years old were removed from the analysis as they failed to reach our minimum accuracy criterion on the hand rotation task (see design and analysis section). The final sample comprised 84 participants, consisting of 12 healthy younger children aged 6–7 years (three males and nine females, *M*_Age_ = 7.13; *SD* = 0.59), 18 healthy older children aged 8–12 (10 males and eight females, *M*_Age_ = 11.15; *SD* = 1.26), 18 age matched 8–12 years old children with pDCD (11 males and seven females, *M*_Age_ = 10.94; *SD* = 1.25), 17 healthy adolescents aged 13–17 years (13 males and four females, *M*_Age_ = 14.87; *SD* = 1.62) and 19 healthy adults aged 19–34 years (13 males and 6 females, *M*_Age_ = 25.45; *SD* = 3.62). These approximate age groups are commonly adopted in MI (Deconinck et al., 2009; Williams et al., 2011a,b) and motor planning research (e.g., Noten et al., 2014; Wilmut and Byrne, 2014b) where participants are grouped according to age. Critically, preliminary analysis failed to show significant correlations between age and our motor planning metric in any of the age groups (*p* ≥ 0.402 for all age groups), suggesting that chronological age was not linked to performance on the planning task within these different age bands. The project received ethical clearance from the relevant university Human Research Ethics Committees and from the Victorian Department of Education and Early Childhood Development (DEECD). Children and adolescents were recruited from two primary schools and two secondary schools in metropolitan Melbourne, Australia. Adults were undergraduate students attending a university in Melbourne, Australia.

All participants completed the McCarron Assessment of Neuromuscular Development (MAND; McCarron, 1997) and a neuromuscular development index (NDI) was calculated. Typically developing participants were considered to have age-appropriate motor skill level, scoring above the 20th percentile on the MAND (i.e., NDI ≥ 90; one participant included scored at the 19th percentile). Children in the pDCD group were screened according to our previously successfully adopted measures, which address DSM diagnostic criteria (see Williams et al., 2008; Hyde and Wilson, 2011a,b, 2013). Children in the pDCD group displayed motor skill levels at or below the 15th percentile (i.e., NDI < 85), suggesting that acquisition and/or execution of motor skills was significantly below that expected given the child’s chronological age (Geuze et al., 2001; American Psychiatric Association [APA], 2013; Criterion A). Where possible, parents and/or school classroom or sporting teachers verbally confirmed the presence of motor-related difficulties in the classroom and/or during physical education (Criterion B), with the onset of motor skill difficulties arising early in development (Criterion C). Exclusion criteria were a prior diagnosis of an intellectual disability, a neurological condition affecting movement (e.g., cerebral palsy, muscular dystrophy) or visual impairment (Criterion D). To control for co-morbid disorders, children were also excluded from the study if they had a prior diagnosis of attention and/or learning difficulties, as reported by parents and/or teachers. Further, since children were recruited from mainstream primary schools they were assumed to have IQ levels within the normal range (Geuze et al., 2001). While children in the pDCD group were, where possible, assessed against the DSM-5 (American Psychiatric Association [APA], 2013) criteria for DCD, in the absence of a full clinical assessment we opted to refer to this group as ‘probable’ DCD (pDCD).

### Measures and Procedure

#### Motor Planning Task

**FIGURE 1:**
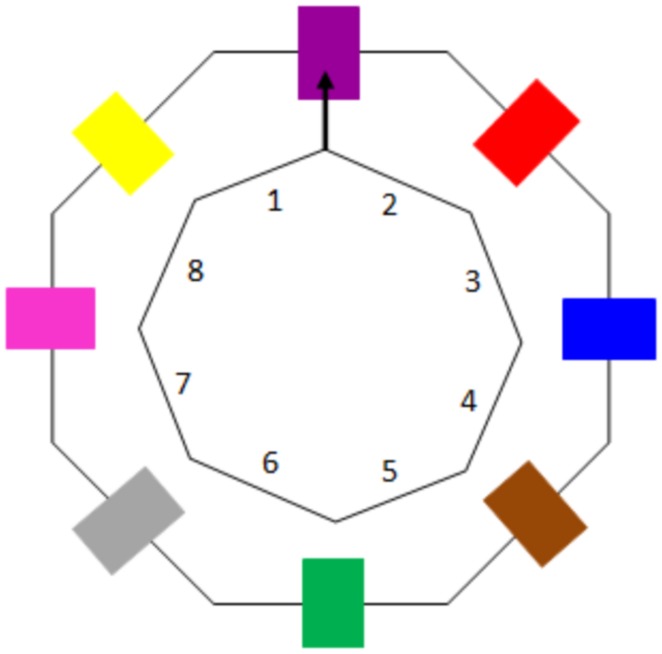
**A schematic illustration of the octagon and surrounding colors, including coding numbers**.

The ability to plan for ESC was assessed using a grip selection task identical to the one described by Wilmut and Byrne (2014a,b). Participants were seated in front of a wooden octagon mounted on a board (see **Figure 1**), which could be rotated so that an arrow (initially pointing at 0° upwards with respect to the participant’s midline) pointed to one of eight peripheral locations, indicated by differently colored stripes located at the center of each of the sides. The size of the octagon varied according to the size of the participant’s hand, ranging from 6.5 to 12.5cm in diameter. A start node was located approximately one third of the participant’s arm length away from the octagon. Trials were recorded using a video camera mounted on a tripod.

Participants began each trial by grasping the start node between their thumb and index finger. The experimenter then called out a color or a sequence of colors and the participant was required to grasp the octagon and rotate it so that the arrow pointed to the designated color. Participants were informed that they were free to grasp the octagon in any way they liked and that they could change their grasp between trials but not during a trial (i.e., after they had grasped the octagon to commence a trial). Participants were advised to place no more than one finger on each of sides of the octagon and instructed to keep their fingers in contact with the octagon during the movement. No explicit instructions asking participants to think about their grasp or raising the issue of comfort were given.

The experiment consisted of 12 one-color sequences, 12 two-color sequences, and 12 three-color sequences (*N* = 36). Color sequences were presented in blocked rather than random fashion (see Wilmut and Byrne, 2014a,b), starting with the one-color sequences and ending with the three color sequences. At the beginning of the task, the experimenter modeled one trial of each sequence length that was not part of the test protocol. For all color sequences, participants were able to rotate the octagon in either clockwise or anticlockwise fashion, or in a combination of these directions for multiple color sequences. For two and three-color sequences, participants rotated the octagon so that the arrow initially pointed to the first color of the sequence, eventually ending the movement by rotating the octagon so that the arrow pointed to the final color of the sequence. All multiple color sequences included a movement to green (i.e., a movement requiring a 180° rotation); one color sequences could include rotations to green, but also included movements to colors requiring less rotation.

#### Hand Rotation Task

The ability to perform MI was assessed using the hand rotation task (Parsons, 1994). Single hand stimuli (9 cm × 8 cm, centerd in the middle of a 13 in. screen), programed with E-Prime software (Version 2.0, Psychology Software Tools, Pittsburgh, PA, USA), were presented on a laptop computer. Participants were seated at a comfortable distance from the screen and instructed to decide whether each stimulus was a left or a right hand as quickly and accurately as possible. Hands were presented randomly in 45° increments between 0 and 360° and remained on screen until a response was recorded by pressing a designated key on the computer keyboard or 10 s had passed. Stimuli were shown in either palm view (palm of the hand facing toward participants) or back view (back of the hand facing toward participant). For each stimulus, we recorded response time (RT) and accuracy; RT was recorded to the nearest 1 ms. Participants completed five practice trials followed by 80 test trials, resulting in eight trials per angle. Participants did not receive specific instructions cueing the use of imagery (motor or otherwise). Thus, while an implicit measure of MI, preliminary analysis of performance profiles in each group verified the use of a MI strategy (see below) prior to age comparisons taking place.

### Design and Analysis

Alpha was set at 0.05 for all statistical analyses and adjusted for multiple comparisons using the Benjamini-Hochberg FDR procedure (Benjamini and Hochberg, 1995, see also Williams et al., 1999). ESC and RT data were screened for values falling outside ±3 SD (no cases were removed). Regression models where checked for influential cases. Cases where Cook’s *D* approached or exceeded 1 were inspected and removed from the analysis if they unduly influenced the model parameters (Stevens, 2002). This led to the removal of two cases (see results section).

#### Motor Planning Task

Based on the video recordings, the proportion of movements ending in ESC was calculated for each participant at each sequence length. Trials where the participant turned the octagon back to the start node and/or trials where the participants did not follow the instructions correctly were excluded from the analysis. This resulted in the exclusion of an average of three (9%) trials per participant in the 6–7 years old age group, one (2%) trial per participant in the 8–12 years old group and less than one trial per participant (<1%) trial in all other groups. As per Wilmut and Byrne (2014a,b), to determine if a participant finished a movement in ESC, we initially recorded the position of the thumb at the start of the movement, using the coding scheme shown in **Figure 1**. For each trial, the start position of the thumb was then assigned a comfort rating based on the previously validated comfort ratings by Wilmut and Byrne (2014a,b). In order to ensure reliability of coding, a second coder coded 15% of participants. Inter-rater reliability analysis (Cohen’s κ) showed excellent agreement between the two raters for 6–7 year olds (κ = 0.95, *p* < 0.001), 8–12 year olds (κ = 0.94, *p* < 0.001), 13–17 year olds (κ = 1.00, *p* < 0.001), 18–24 year olds (κ = 0.98, *p* < 0.001) and the pDCD group (κ = 0.97, *p* < 0.001). A movement was deemed to end in ESC if the assigned comfort rating was less than two (see Wilmut and Byrne, 2014a,b). To investigate the effect of age on the ability to plan for ESC, linear and higher order regression models were fitted to the ESC data. The mean proportion of movements ending in ESC was used as the dependent variable, while linear and higher order effects of age were entered as predictors into the model (see Aiken and West, 1991). To test for group differences between 8 and 12 years old children with and without pDCD to plan for ESC, a one-way ANOVA was run with mean ESC as the dependent variable and group as the independent variable.

To investigate potential biases for grip selection, the position of the thumb was recorded on trials that did not end in ESC. Specifically, similar to previous research investigating grip selection strategies (van Swieten et al., 2010; Wilmut and Byrne, 2014a), we wanted to test for a minimal rotation (MR) bias whereby a participants’ grip selection was based on minimizing or preventing initial wrist rotation from the start node to the octagon at the expense of achieving ESC rather than selecting a grip that would require greater initial wrist rotation but allow the movement to end in comfort [note that while van Swieten et al. (2010) classified movements as biased by a MR strategy based on MR of the to be grasped object, both classification procedures are similar as they reflect minimal effort at the start of the movement at the expense of a comfortable end-position]. Accordingly, similarly to Wilmut and Byrne (2014a), we classified movements that did not end in ESC and where the participant placed their thumb on nodes 6 or 7 (right handers) or on nodes 4 or 5 (left handers) as being biased by a MR strategy and then calculated the proportion of these trials. To investigate the effect of age on MR bias, linear and higher order regression models were fitted to the MR data. The mean proportion of movements where participants used a MR strategy was used as the dependent variable, while linear and higher order effects of age were entered as predictors into the model. To test for group differences between 8 and 12 years old children with and without pDCD to use a MR strategy, a one-way ANOVA was run with mean MR as the dependent variable and group as the independent variable.

#### Hand Rotation Task

For each participant, Mean RT (using both correct and incorrect trials, see Fuelscher et al., 2015a,b) and accuracy for each hand at each angle of rotation was calculated. Trials with RTs less than 250 ms were deleted from the analysis, resulting in the inclusion of an average of 77 (96%) valid trials for 6–7 year olds, 79 (99%) for 8–12 year olds, 13–17 year olds and adults, respectively. Mean accuracy was calculated as the proportion of correct responses over all trials. To ensure that participants were capable of differentiating hand laterality at the most basic level of stimulus presentation (see Butson et al., 2014), a 60% accuracy criterion for hands presented at 0° was set as a minimum requirement to include the data in the analysis. This criterion resulted in the exclusion of eight 6–7 year olds (50%), one healthy 8–12 years old (1%), one 8–12 years old with pDCD (1%), and one 13–17 years old (1%) from the initial sample of 95 participants.

To confirm that performance of participants was constrained by the biomechanical constraints of action, separate two-way mixed-design ANOVAs on RT then on accuracy were run with group (i.e., 6–7, 8–12, 13–17, 18–34, pDCD) as the between subjects factor, and with either direction of rotation (i.e., medial vs. lateral) or stimulus view (i.e., back vs. palm) as the within subjects factor. Medial rotation performance was calculated as the average of responses for left hands presented at 45, 90, and 135° and right hands presented at 315, 270, and 225°. Lateral rotation performance was calculated as the average responses for left hands presented at 315, 270, and 225° and right hands presented at 45, 90, and 135°. For each stimulus view condition (i.e., back vs. palm), performance was averaged across angular rotations of 0, 45, 90, 135, and 180°. In line with previous research (see Butson et al., 2014), general hand rotation performance was analyzed by collapsing medial and lateral rotations and by combining palm and back views to provide mean values for responses from 0 and 180° (45° increments; eight trials per angle).

To confirm whether mean RT and mean accuracy (i.e., averaged across angular rotation of 0, 45, 90, 135, and 180°) provided a valid gross measure of imagery performance on the hand rotation task, both RT and accuracy were submitted to a one-way repeated measures ANOVA with angle as the repeated measures factor. Age was entered as a covariate to test if the effect of angle varied according to age. To confirm that mean RT and accuracy (i.e., averaged across angular rotation) provided a valid gross measure of MI, analysis needed to demonstrate a constant association between angular rotation and age, that is, a non-significant interaction effect. To investigate the effect of age on MI ability, linear and higher order regression models were fitted to the RT and accuracy data. RT and accuracy from the hand rotation task were used as the dependent variable, respectively, linear and higher order effects of age were entered as predictors into the model. To test for group differences between 8 and 12 years old children with and without pDCD on MI ability, a one-way ANOVA was run with group as the independent variable and RT and accuracy as the dependent variables, respectively.

#### Developmental Association Between Motor Planning and MI Ability

##### Typically developing children and adults

To determine if performance on the MI task was a significant predictor of the ability to plan for ESC in typically developing children and adults (note that separate regression analyses were conducted for children with pDCD), a hierarchical regression was conducted with ESC as the dependent variable and RT and accuracy on the hand rotation task as predictors at Step 1. Linear and higher order effects of age were then entered as predictors at Step 2 to investigate whether the developmental progression of end-state planning was influenced by general age-related neuro-motor improvements over and above those accounted for by action representation (see Aiken and West, 1991). The inclusion of age into the model was also a necessary prerequisite for the final model that was designed to test if the association between action representation (i.e., MI performance) and the ability to plan for ESC varied according to age. To this end, we created a moderating term representing the interaction between ESC and age (see Aiken and West, 1991). Predictors were mean centered prior to analysis to allow for an interpretation of the interaction term.

##### Children aged 8–12 years with pDCD

A multiple regression was run with RT and accuracy on the hand rotation task as predictors and ESC as the dependent variable to investigate if action representation (i.e., MI) significantly predicted the ability to plan for ESC in children with pDCD. This analysis was considered exploratory given the modest sample size. However, we felt that an inspection of the multiple correlation coefficient (i.e., our measure of effect size) would nevertheless provide valuable information on possible trends with respect to the direction and strength of the association.

## Results

### Developmental and Motor Skill Comparisons of Grip Selection Planning

**FIGURE 2:**
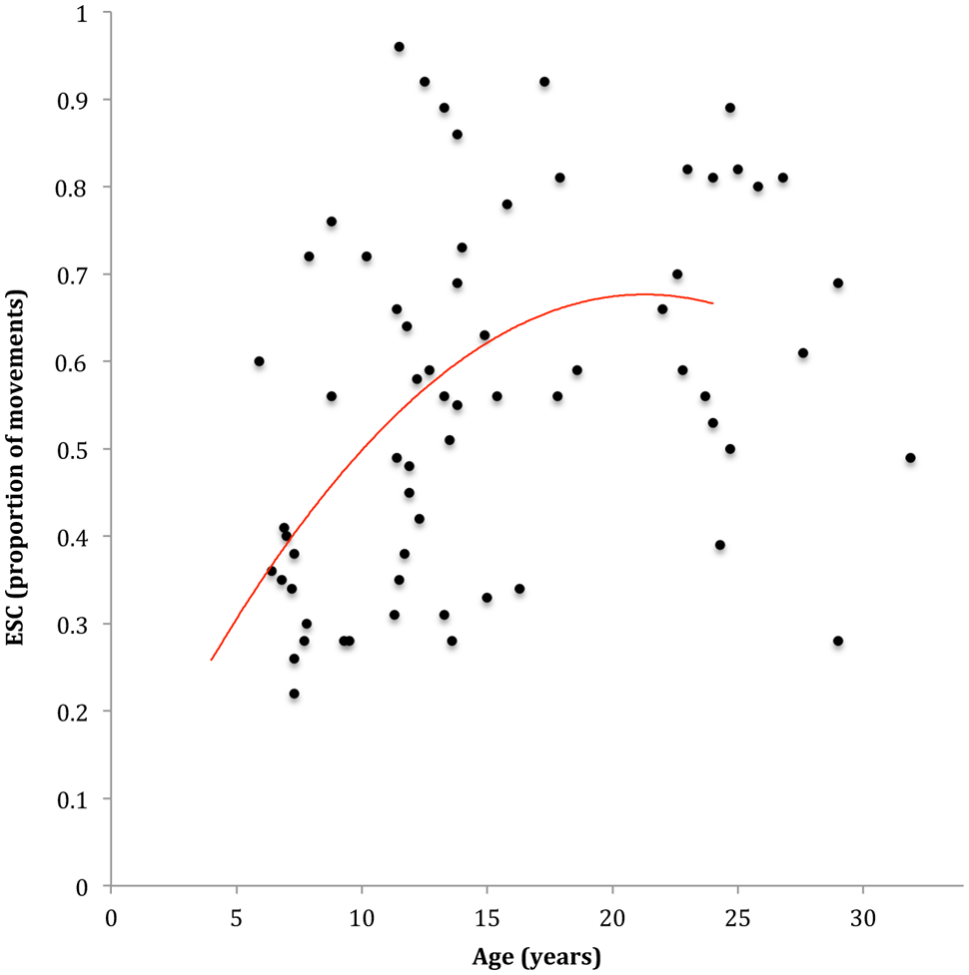
**Scatterplot and line of best fit modeling the association between age and end-state comfort (ESC)**.

Participants finished significantly more movements in end-state comfort on one color sequences (*M*_ESC_ = 0.60, *SD* = 0.21) than on two color sequences (*M*_ESC_ = 0.50, *SD* = 0.24), *p* < 0.001, $\eta_{p}^{2}$ = 0.27. No significant difference were observed between two and three (*M*_ESC_ = 0.48, *SD* = 0.23) color sequences, *p* = 0.414, $\eta_{p}^{2}$ = 0.01 and no significant interaction was found with age. The regression analysis modeling the association between age and ESC is presented in **Figure 2**. One case was excluded from the analysis due to excessive influence. Results indicated that a quadratic association between age and mean ESC provided the model with the best fit, explaining approximately 22% of the variability in ESC, *F*(2,62) = 8.69, *p* < 0.001, *AdjR*^2^ = 0.19. Specifically, the tendency to plan for ESC showed comparatively sharper increases through the 6–12 years span, with more subtle increases thereafter (see **Figure 2**). The one-way ANOVA showed that 8–12 years old children with pDCD (*M*_ESC_ = 0.39, *SD* = 0.13) finished significantly less movements in end-state comfort than their typically developing peers (*M*_ESC_ = 0.55, *SD* = 0.20), *F*(1,34) = 7.19, *p* = 0.011, $\eta_{p}^{2}$ = 0.18.

**FIGURE 3:**
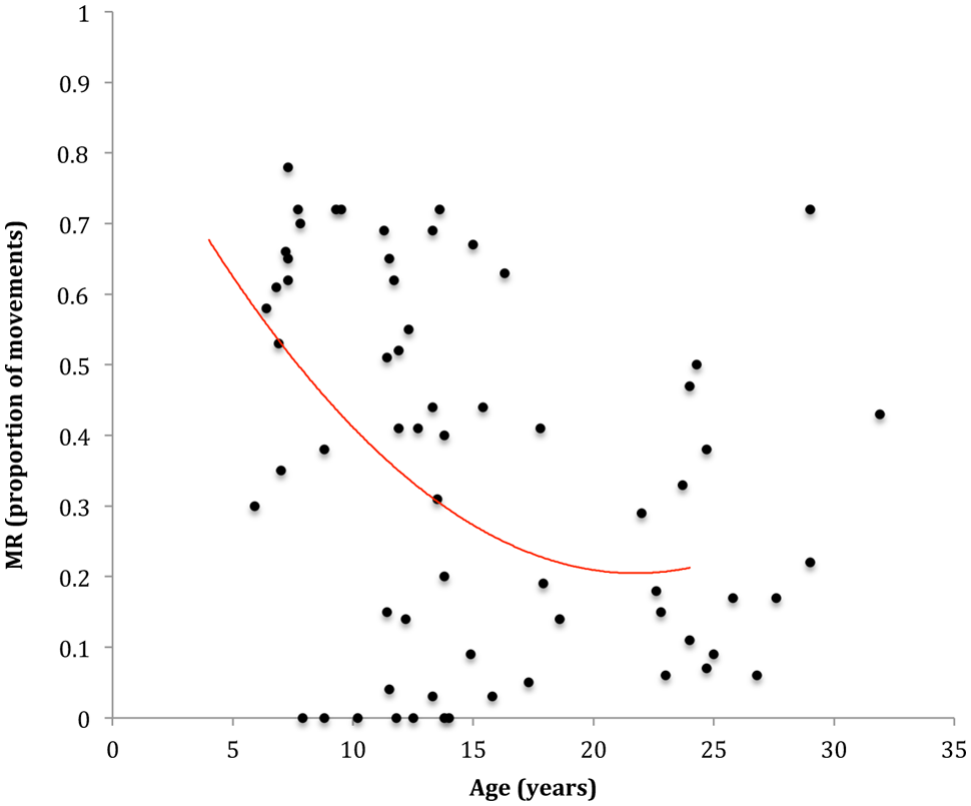
**Scatterplot and line of best fit modeling the association between age and MR**.

Participants used a MR strategy more often on two color sequences (*M*_MR_ = 0.40, *SD* = 0.29) than on one color sequences (*M*_MR_ = 32, *SD* = 0.25), *p* < 0.001, $\eta_{p}^{2}$ = 0.18. No significant difference was observed between two and three color sequences (*M*_MR_ = 0.41, *SD* = 0.30) and no significant interaction was found with age. The regression analysis modeling the association between age and MR is presented in **Figure 3**. One case was excluded from the analysis due to excessive influence. Results indicated that a quadratic association between age and mean MR provided the model with the best fit, explaining approximately 16% of the variability in MR, *F*(2,62) = 6.06, *p* = 0.004, *AdjR*^2^ = 0.14. Specifically, the tendency to use a MR strategy decreased substantially from 6 to 12 years, with more subtle decreases thereafter (see **Figure 3**). The one-way ANOVA showed no significant differences between 8 and 12 year olds with (*M*_MR_ = 0.49, *SD* = 0.22) and without pDCD (*M*_MR_ = 0.36, *SD* = 0.28). Results are presented in **Figure 3**.

### The Development of Motor Imagery: Hand Rotation Performance

#### Biomechanical Effects

Mean values for all biomechanical constraints analyses are presented in **Table 1**. The two (direction of rotation: medial vs. lateral) by five (group) mixed ANOVA on RT showed a significant main effect for direction of rotation, Wilk’s Λ = 0.54, *F*(1,79) = 68.12, *p* < 0.001, $\eta_{p}^{2}$ = 0.46, and a significant main effect for group, *F*(4,79) = 20.35, *p* < 0.001, $\eta_{p}^{2}$ = 0.51. No significant interaction effect was observed. Averaged across group, participants responded faster to medially rotated stimuli (1979 ms, *SD* = 774 ms) than to laterally rotated stimuli (2342 ms, *SD* = 929 ms). The two (direction of rotation: medial vs. lateral) by five (group) mixed ANOVA on accuracy showed a significant interaction effect, Wilk’s Λ = 0.78, *F*(4,79) = 5.14, *p* = 0.001, $\eta_{p}^{2}$ = 0.21. The interaction effect suggested that while participants responded more accurately to medially rotated stimuli than to laterally rotated stimuli (see **Table 1**), this difference was less pronounced in adolescents and adults and failed to reach significance in any of the groups after correcting for multiple comparisons.

**Table 1:** Descriptive statistics for biomechanical constraints analyses in each age group.

	6–7 (*N* = 12)		8–12 (*N* = 18)		13–17 (*N* = 17)		18–34 (*N* = 19)		pDCD (*N* = 18)	
	RT	ACC	RT	ACC	RT	ACC	RT	ACC	RT	ACC
MR	2934 (927)	0.83 (0.13)	1973 (568)	0.94 (0.07)	1570 (382)	0.94 (0.05)	1340 (456)	0.94 (0.07)	2408 (479)	0.90 (0.12)
LR	3471 (1032)	0.62 (0.26)	2321 (657)	0.90 (0.11)	1873 (593)	0.93 (0.06)	1608 (549)	0.93 (0.07)	2830 (686)	0.85 (0.11)
BV	2991 (894)	0.81 (0.17)	2016 (439)	0.92 (0.07)	1561 (370)	0.92 (0.06)	1465 (535)	0.92 (0.06)	2446 (567)	0.90 (0.07)
PV	3384 (859)	0.62 (0.20)	2344 (708)	0.89 (0.12)	1999 (453)	0.91 (0.09)	1708 (553)	0.92 (0.08)	2921 (523)	0.84 (0.16)

*MR, medial rotation; LR, lateral rotation; BV, back view; PV, palm view; RT, mean response time (ms); ACC, mean accuracy (proportion correct); *SD* in brackets*.

The two (stimulus view: palm vs. back) by five (group) mixed ANOVA on RT showed a significant main effect for direction of rotation, Wilk’s Λ = 0.43, *F*(1,79) = 103.86, *p* < 0.001, $\eta_{p}^{2}$ = 0.57, and a significant main effect for group, *F*(4,79) = 20.34, *p* < 0.001, $\eta_{p}^{2}$ = 0.51. No significant interaction effect was observed. Averaged across group, participants responded faster to stimuli presented in back view (2031 ms, *SD* = 767 ms) than to stimuli presented in palm view (2402 ms, *SD* = 839 ms). The two (stimulus view: palm vs. back) by five (group) mixed ANOVA on accuracy showed a significant interaction effect, Wilk’s Λ = 0.84, *F*(4,79) = 3.87, *p* = 0.006, $\eta_{p}^{2}$ = 0.16. The interaction effect indicated that though participants responded more accurately to stimuli presented in back view than to stimuli presented in palm view (see **Table 1**), this difference was less pronounced in adolescents and adults and failed to reach significance in any of the groups after correcting for multiple comparisons.

#### Age and Motor Skill Effects

**FIGURE 4:**
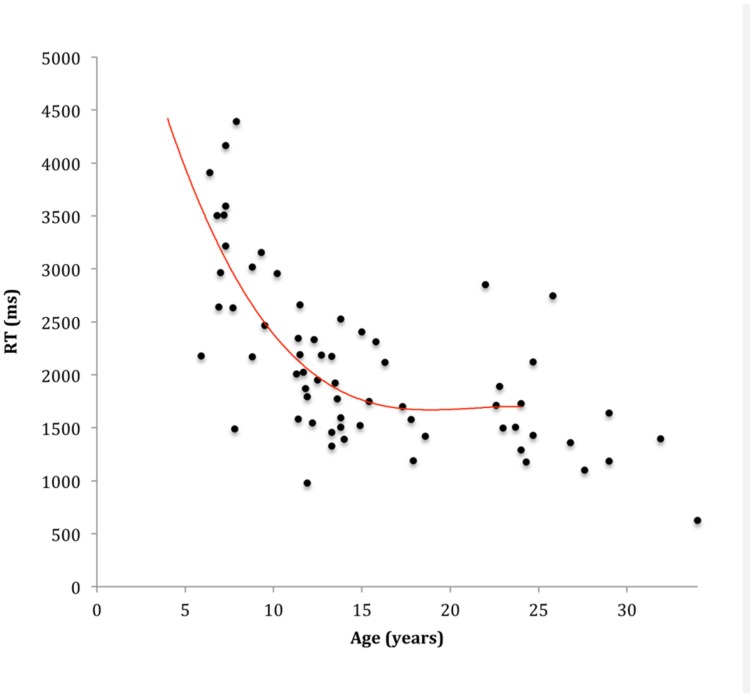
**Scatterplot and line of best fit modeling the association between age and response time (RT) on the hand rotation task**.

The repeated measures ANOVA on RT revealed no significant interaction effect between angular rotation and age. Accordingly, mean RT provided a valid gross measure of imagery performance on the hand rotation task. The regression analysis modeling the association between age and RT is presented in **Figure 4**. Results indicated that a cubic association between age and mean RT provided the model with the best fit, explaining approximately 47% of the variability in ESC, *F*(3,62) = 25.21, *p* < 0.001, *AdjR*^2^ = 0.45. Specifically, the extent of RT reduction with age was greatest from 6 to 12 years, with more subtle decreases observed thereafter (see **Figure 4**). The one-way ANOVA indicated that 8–12 years old children with pDCD (*M* = 2683 ms, *SD* = 513 ms) were slower to respond than their typically developing peers (*M* = 2181 ms, *SD* = 552 ms), *F*(1,34) = 8.03, *p* = 0.008, $\eta_{p}^{2}$ = 0.19.

**FIGURE 5:**
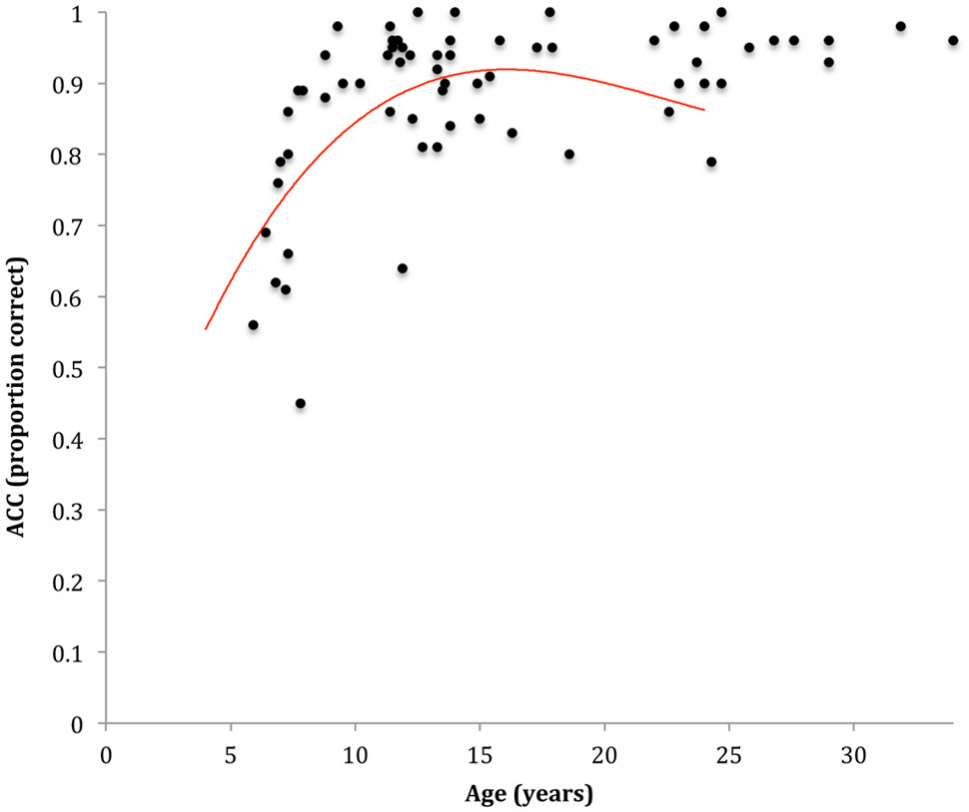
**Scatterplot and line of best fit modeling the association between age and accuracy (ACC) on the hand rotation task**.

The repeated measures ANOVA on accuracy revealed no significant interaction effect between angular rotation and age. Accordingly, mean accuracy provided a valid gross measure of imagery performance on the hand rotation task. The regression analysis modeling the association between age and accuracy is presented in **Figure 5**. Results indicated that a cubic association between age and mean accuracy provided the model with the best fit, explaining approximately 43% of the variability in ESC, *F*(3,62) = 15.44, *p* < 0.001, *AdjR*^2^ = 0.40. Specifically, accuracy increased substantially from 6 to 12 years, with more subtle increases observed thereafter (see **Figure 5**). The one-way ANOVA indicated no significant differences in accuracy between 8 and 12 years old children with pDCD (*M* = 0.87, *SD* = 0.09) and their typically developing peers (*M* = 0.91, *SD* = 0.08), *p* = 0.208, $\eta_{p}^{2}$ = 0.05.

### The Association Between Motor Imagery and Grip Selection Planning

#### Typically Developing Children and Adults

While our results suggested that a quadratic association provided the model with the best for the association between age and ESC, a cubic association was found for the association between age and MI performance. As such, the long-term developmental trajectories for these associations can be expected to differ. That is, ESC performance can be expected to decrease in an exponential fashion while MI performance can be expected to increase. However, as can be seen in **Figures 2** and **5**, the main difference between the trajectories was a sharper improvement for MI performance early in development compared to ESC, while the overall shape of these trajectories was similar from childhood to early adulthood. Accordingly, modeling a linear association between MI and ESC is appropriate across the age range in this study.

Results of the three-step hierarchical regression are displayed in **Table 2**. The initial model demonstrated that, in combination, RT and accuracy (from the hand rotation task) were significant predictors of ESC, accounting for approximately 21% (Δ*R*^2^ = 0.21, *p* = 0.001) of the variability in ESC. Individually, accuracy (*sr* = 0.34, *p* = 0.003) but not RT (*sr* = -0.19, *p* = 0.102) made significant unique contributions to ESC. The inclusion of age in the model did not significantly improve prediction of ESC (Δ*R*^2^ = 0.02, *p* = 0.238), adding only 2% of explained variability. Notably, accuracy (*sr* = 0.27, *p* = 0.018) continued to make a significant unique contribution to ESC even after age had been accounted for in the model. No significant higher order effects were found for the association between age and ESC at this step. Accordingly, only the linear effect of age was included in the final model.

**Table 2:** Summary of regression analysis for variables predicting ESC (*N* = 66).

	Model 1			Model 2			Model 3		
Variable	*B*	*SE*	β	*B*	*SE*	β	*B*	*SE*	β
RT	0.00	0.00	-0.20	0.00	0.00	-0.10	0.00	0.00	-0.07
ACC	0.65	0.21	0.36^∗∗^	0.55	0.23	0.31^∗^	0.89	0.34	0.50^∗^
Age				0.04	0.05	0.18	0.04	0.05	0.11
RT × Age							0.00	0.00	0.14
ACC × Age							0.07	0.04	0.27
*AdjR*^2^		0.19			0.19			0.20	
*F*_change_		8.53^∗∗^			1.42			1.41	

*RT, response time; ACC, Accuracy. RT, ACC, and Age were centered at their means. ^∗^*p* < 0.05, ^∗∗^*p* < 0.01*.

The purpose of the final model, which included age as a potential moderator, was to investigate if the association between imagery performance and ESC varied according to age. Results demonstrated that age did not significantly moderate the associations between imagery performance and ESC (Δ*R*^2^ = 0.04, *p* = 0.252). Neither the interaction term for RT and age, nor the interaction term for accuracy and age made a significant unique contribution to the prediction of ESC.

#### Children Aged 8–12 with pDCD

Response time and accuracy (from the hand rotation task) did not significantly predict ESC in children with pDCD. Indeed, the model explained less than 3% of the variability in ESC.

## Discussion

Previous research has demonstrated that a child’s ability to plan grasp selection matures in a non-monotonic fashion (Stöckel et al., 2012; Scharoun and Bryden, 2014; Wilmut and Byrne, 2014a; Wunsch et al., 2014). This study aimed to investigate the degree to which this profile of development is associated with the quality of internal action representations. Consistent with earlier accounts, we showed that, for typically developing individuals, the proficiency of grip selection planning developed along a non-linear trajectory. Indeed, substantial improvement was observed from 6 to 12 years (measured as the percentage of grip-selection trials terminating in ESC) and more subtle improvements thereafter into adolescence and adulthood. Children with pDCD showed atypical grip selection planning, indicated by a reduced tendency to plan for ESC compared to their age-matched peers. Analysis of performance on the hand rotation task suggested a similar, although not identical, profile for the development of children with pDCD’s ability to engage internal action representations.

Critically to the present paper, regression analysis confirmed our hypothesis that for typically developing individuals, the abovementioned developmental profile of improving efficacy of grip selection planning would be associated with a greater capacity to engage internal action representations. Specifically, the strength of the ESC effect (i.e., the percentage of trials terminating in ESC) during the grip selection task was predicted by participants’ performance on the hand rotation task. This profile of association remained stable throughout typical development suggesting that the influence of internal action representation on grip selection planning remained constant with age. Our data support computational accounts of motor planning, which suggest that internal action representations are associated with both the expression and development of mature grip selection planning. Interestingly, no such association was found for our atypically developing (i.e., pDCD) children. We argue that this finding provides preliminary evidence that children with pDCD may be less reliant on internal action representations than their age-matched control counterparts when planning their grasping movements. Taken together, our findings are an important step toward understanding the neuro-cognitive mechanisms that contribute to grip-selection development and its pathology. These are discussed below.

### The Development of Grip Selection Planning

With respect to the influence of sequence length on ESC during the grip selection task, our results showed that participants demonstrated a greater tendency to plan for ESC on one-sequence movements compared to two and three sequence movements. Our findings are thus broadly similar to those of Wilmut and Byrne (2014a,b) who showed that the propensity to opt for ESC was greatest on easier (i.e., one-sequence) movements during grip selection. Further, our results were consistent with the previously reported non-linear development of grip selection planning across the primary school years and into adulthood (Stöckel et al., 2012; Wilmut and Byrne, 2014a; Wunsch et al., 2014). Specifically, inspection of the developmental trajectory suggested that the tendency to plan for ESC increased markedly from 6 to 12 years, with improvement becoming less pronounced into adolescence and adulthood. Since computational modeling suggests that optimal grip selection planning is dependent on one’s ability to effectively generate and/or engage internal action representations (Rosenbaum et al., 1995, 2001, 2014; Johnson, 2000; Glover, 2004), we argue that this non-monotonic maturation may be subserved, at least partly, by a greater capacity to generate and/or engage internal action representations.

In support of the above view, we observed that the development of the ESC effect was paralleled by a decreasing tendency to opt for a MR strategy. That is, while younger children favored a MR strategy, older participants were less likely to adopt this strategy. A similar developmental pattern has been reported in earlier studies of grip selection planning (van Swieten et al., 2010; Wilmut and Byrne, 2014a). Since MR reflects the tendency to favor a MR strategy often at the expense of ending the movement comfortably, it has been argued that the propensity for younger children to opt for MR may indicate that they are less aware of the biomechanical costs associated with a MR strategy (see van Swieten et al., 2010). Since the sensory consequences of a movement’s terminal position are considered to constitute part of the hierarchy of costs that influence the selection of a movement plan (Rosenbaum et al., 1995, 2001; Stöckel and Hughes, 2015b), it may be that younger children favor a MR strategy because they are less able to generate and/or engage internal action representations to predict the sensory consequences of their movements (see below). This suggestion is consistent with the abovementioned view that the rapid improvement in planning efficiency observed during the primary school years (i.e., 6–12 years) reflect, at least in part, an improved capacity to engage said action representations. We elaborate on this hypothesis below.

In line with previous work (van Swieten et al., 2010; Wilmut and Byrne, 2014b), children with pDCD demonstrated an atypical pattern of grip selection planning relative to their age-matched control peers. Specifically, children with poor motor skill were less likely to opt for ESC than their age matched peers. While these results are consistent with previous evidence reporting that action planning may be less efficient in children with DCD (van Swieten et al., 2010; Wilmut and Byrne, 2014b), some work has failed to replicate this effect (Smyth and Mason, 1997; Noten et al., 2014). As noted earlier, however, where group differences have not been reported as per Noten et al. (2014), a ceiling effect has been evident on the grip selection task adopted to measure grasp planning. This might have reduced the power of the design and hence decreased the likelihood of detecting meaningful group differences in grip selection planning between DCD and control groups should they exist.

### The Development of Internal Action Representations

Consistent with the use of an embodied MI strategy, participants responded slower and less accurately to physically awkward (i.e., lateral) rotations, than to physically comfortable (i.e., medial) rotations (e.g., Toussaint et al., 2013; Noten et al., 2014). Similarly, participants responded slower and less accurately when hands were presented in the posturally incongruent palm view than when hands were presented in the posturally congruent back view (e.g., Butson et al., 2014; Hyde et al., 2014). Notably, with respect to accuracy, these effects failed to reach significance at the individual group level after controlling for multiple comparisons. This was not surprising, however, given that accuracy performance approached ceiling in older children, adolescent and adults, a finding that has previously been reported (e.g., Hyde et al., 2013). Thus, we argue that the biomechanical and postural performance effects provide qualified evidence that typically developing individuals 6 years and above and atypically developing individuals were engaged in a MI strategy to perform the hand rotation task.

Similarly to earlier developmental work (Caeyenberghs et al., 2009; Smits-Engelsman and Wilson, 2013), the ability of typically developing children to engage in MI improved substantially from 6 to 12 years with only subtle improvements observed thereafter into adolescence and adulthood. This general pattern of development was observed for both RT and accuracy data on the hand rotation task. These results are consistent with earlier suggestions that the ability for healthy individuals to generate and/or engage internal action representations improves substantially during the primary school years, and incrementally henceforth. Consistent with previous work investigating hand rotation performance in individuals with DCD (e.g., Williams et al., 2008; Deconinck et al., 2009), 8–12 years old children with pDCD performed slower than their age matched peers. Interestingly, however, children with pDCD performed at similar (though slightly less accurate; $\eta_{p}^{2}$ = 0.05) levels, a trend previously reported (e.g., Williams et al., 2008). As has been proposed previously, we argue that the slower MI performance observed by the pDCD group here may reflect a reduced capacity to generate and or monitor internal action representations (Gabbard and Bobbio, 2011; Adams et al., 2014).

Importantly, the developmental profile of MI and decreased performance observed in individuals with pDCD reported in this section largely mirrors that observed for grip selection planning in the present cohort. While this evidence supports earlier studies on the development of grip selection planning and action representation, respectively, ours is the first to measure both in the same cohort of individuals across this critical developmental spectrum. Foremost to the aim of this study, the noted parallel developmental profiles for each lend preliminary support to the view that internal action representations may be associated with the development and expression of grip selection planning. The following section discusses the findings of a statistical test of this hypothesis.

### Maturation of Grip Selection Planning is Associated with Improved Action Representations Across Typical Development but not in Children with pDCD

The initial step of the regression model for typically developing individuals demonstrated that faster and more accurate performance on the hand rotation task predicted a greater tendency to opt for ESC on the grip selection task. In combination RT and accuracy performance on the hand rotation task predicted around 20% of the variability in the percentage of trials terminating in ESC. Broadly speaking, our results provide important support for computational models of motor planning which suggest that optimal grip selection planning may be associated with one’s capacity to implement the action representations (Rosenbaum et al., 1995, 2001; Johnson, 2000; Glover, 2004). Importantly, this work extends previous research using young children (Toussaint et al., 2013) by showing that this association exists across the full developmental spectrum from early childhood into adulthood. Interestingly, accuracy but not RT made a significant unique contribution to predicting variability in ESC, which was surprising given the largely parallel developmental profile of RT and ESC effect across typical development. While we did observe a non-significant trend (*p* = 0.102) for shorter RTs to be associated with an increased tendency to plan for end-state comfort, it appears that despite the parallel developmental improvements on our motor planning and MI measures at a group level, this association might either be more variable at the individual level or might not be constant across age. Accordingly, RT was kept in the model to test for a possible interaction of RT with age in predicting ESC.

The inclusion of age as a predictor did not significantly improve the model, adding only 2% of explained variability over and above that predicted by hand rotation performance. This, however, likely reflected the fact that much of the variability accounted for by age in predicting the ESC effect had already been accounted for by developmental improvements in MI ability. Notably, however, accuracy continued to make a significant unique contribution to the prediction of ESC even after age had been accounted for in the model.

Critical to the present study, the final step of the model, which included age as a potential moderator of the association between hand rotation performance and the ESC effect, showed that neither the interaction term for accuracy nor the interaction term for RT made significant unique contributions to the prediction of the strength of the ESC effect. This evidence suggests that the association between hand rotation performance and ESC stayed largely constant across the age range in our present sample. Accordingly, it appears that nature of the association between internal action representations and the expression of grip-selection planning does not alter significantly as a function of age. Taken together, the final model provides the first available empirical evidence in support of the view that the non-linear developmental improvements in the proficiency with which typically developing individuals plan grip selection that we observed (and has been reported previously) may be, at least in part, associated with a greater capacity to generate and/or engage internal action representations.

Interestingly, we failed to replicate the above association between the proficiency of grip selection planning and the efficiency of internal action representations implementation in atypically developing children. That is, similarly to Noten et al. (2014), we failed to observe a significant association between response time and accuracy on the hand rotation task and the tendency to opt for ESC when 8–12 years old children with pDCD were considered independently. Indeed, the mean RT and accuracy on the hand rotation task explained less than 3% of the variability in ESC. While we must be circumspect when interpreting this result in light of the modest sample size, this finding was nevertheless unexpected given the parallel sub-optimal grip selection and MI performance, which our group comparisons revealed in our sample of children with pDCD earlier. A possible explanation for these results, however, may be that our children with pDCD were less reliant on internal action representations to plan their grip selection than their typically developing peers, instead opting for an alternative, albeit less successful, strategy. Specifically, research suggests that the integrity of internal action representations is influenced by the certainty with which individuals are able to predict the sensory consequence of action (e.g., Körding and Wolpert, 2004) and prone to the influence of visuomotor experience (Redding et al., 2005). For children with pDCD, whose motor experience is largely atypical, it may be that these children are less certain (i.e., less confident) in engaging internal action representations and consequently develop compensatory strategies less reliant on this system during action planning/execution. Indeed, our results showed that individuals with pDCD were less inclined to terminate grip selection task movement in ESC, instead showing a stronger preference for a MR strategy. Rather than requiring children to mentalize the entire movement when planning their initial grasp (as per an ESC strategy), the MR strategy instead only requires them to consider the beginning of the movement placing comparatively fewer demands on internal action representations. The use of such an alternative strategy by children with pDCD would explain why we observed parallel atypical performance in grip selection planning and action representation in our sample of children with pDCD, yet an absence of a direct association between the two for the pDCD group.

### Implications and Limitations

The key implications of the present study are twofold. Firstly, our study has provided critical empirical evidence that the previously documented non-linear improvements in reach to grasp planning that typify typical development are associated with an increasing capacity to generate and/or engage internal action representations. Further, while our work here confirms earlier findings from a single study in 6 and 8 years old typically developing children, which demonstrated that a child’s ability to implement internal action representations is associated with the proficiency with which they are able to plan grasping actions (Toussaint et al., 2013), our work extends this earlier study by demonstrating that this association holds across the typical developmental spectrum (at least from primary school until adulthood). In doing so, our data support neuro-computational modeling of reach- to-grasp motor planning which suggests that internal action representations are critical to the development and expression of grasp selection planning (Rosenbaum et al., 1995, 2001, 2014; Johnson, 2000; Glover, 2004).

Secondly, our data suggest that children with poor motor skill may adopt an alternative strategy to plan their grasp selection planning. That is, we failed to replicate the observed association between the integrity of internal action representations and reach to grasp planning shown across typical development in children with pDCD. As we have argued, this may be due to children with DCD placing less emphasis on using internal action representations to plan grasping actions than typically developing individuals. Why this might be the case is not entirely clear, though given that our pDCD group showed poorer performance on the hand rotation task than controls, this strategy may be a compensatory outcome designed to account for decreased efficiency in processing when generating and/or engaging action representations. Furthermore, the suggestion that disruption at the level of internal action representations may explain, at least in part, motor control and performance difficulties in DCD has previously been posited by the current authors (Williams et al., 2006, 2008; Hyde and Wilson, 2011a,b; Hyde et al., 2014; Wilmut and Byrne, 2014b) as well as a growing group of external researchers (see Wilson et al., 2013 for a recent review).

While our data provide compelling evidence that grip selection planning is associated with the generation and/or implementation of internal action representations, performance would likely also be affected by other factors. This suggestion is supported by the fact that while MI performance accounted for a significant proportion of variability in the percentage of trials terminating in ESC on the grip selection task, there was nonetheless a substantial proportion of variability in performance that MI performance did not explain. Indeed, while computational modeling suggests that an evaluation of the biomechanical costs associated with a movement’s terminal position (and hence their prediction via internal action representations) is critical to the selection of an efficient movement plan, it also emphasizes the importance of evaluating other cost constraints (e.g., spatial error and travel costs) in the selection of efficient grasp postures (see Rosenbaum et al., 1995, 2001). Further, based on the results of the present study, it is not possible to infer a causal association between the development of internal action representations and the development of reach to grasp planning. Indeed, evidence suggests that the development of MI and anticipatory planning may also be influenced by the development of general cognitive factors such as visuo-spatial working memory ability (Paus, 2005; Stöckel and Hughes, 2015a). Research further indicates that the development of cognitive representations for grasp postures in long term memory (see Stöckel et al., 2012) and executive planning abilities (Stöckel and Hughes, 2015a) may influence anticipatory motor planning. Accordingly, to better understand the factors that may contribute to the observed variability in grip selection during reach to grasp tasks, future research should focus on the influence of different cost constraints on grip selection (i.e., on the selection of a movement plan) and the degree to which the development of individual cognitive capacities (e.g., working and long-term memory structures) may underlie the tendency to plan for ESC across development.

Further, in light of evidence that some children with DCD present with joint hypermobility syndrome (JHS; see for example Kirby and Davies, 2007), it is possible that the presence of this condition might have influenced grip selection for some of the children in the pDCD group. For example, some grip selections that would have been considered borderline uncomfortable for the control group might not have been considered uncomfortable for hypermobile children with pDCD, given an increased range of joint-movement (Kirby and Davies, 2007). However, since prevalence rates suggest that only around one third of children in the pDCD group would be expected to show symptoms of JHS (Kirby and Davies, 2007), and given that this condition would only have affected performance on trials where these children chose a grip that fell just outside the comfortable range, it is unlikely that these effects, had they been present, would have unduly influenced the results.

## Conclusion

Our study has provided empirical evidence that the previously documented non-monotonic improvements in grip selection planning that typify development from the primary school years through to early adulthood may be associated with a greater capacity to generate and/or engage internal action representations. Our study also highlighted that while this association was consistent across the typical developmental age spectrum, it does not seem hold for atypically developing children (pDCD). We argue that children with poor motor skill may adopt an alternative (and apparently sub-optimal) strategy to plan their grasping actions that places less demands on internal action representations. Taken together, these findings are critical to our understanding of the cognitive mechanisms that are associated with expression and development of motor planning, as well as its pathology.

## Author Contributions

All authors listed, have made substantial, direct and intellectual contribution to the work, and approved it for publication.

## Conflict of Interest Statement

The authors declare that the research was conducted in the absence of any commercial or financial relationships that could be construed as a potential conflict of interest.
